# Design and Synthesis of Redox-Switched Lariat Ethers and Their Application for Transport of Alkali and Alkaline-Earth Metal Cations Across Supported Liquid Membrane

**DOI:** 10.1155/BCA/2006/97141

**Published:** 2006-08-16

**Authors:** Anubhuti Awasthy, Mamta Bhatnagar, Jyoti Tomar, Uma Sharma

**Affiliations:** School of studies in Chemistry, Vikram University, Ujjain 456010, India

## Abstract

A new class of redox-switched anthraquinone derived lariat ethers 1-(1-anthraquinonyloxy) 3, 6, 9 trioxaundecane 11-ol (M_1_), 1-(1-anthraquinonyloxy) 3, 6 dioxaoctane 9-ol (M_2_), 1-(1-anthraquinonyloxy) 3 oxapentane 5-ol (M_3_), 1-(1-anthraquinonyloxy) 3 oxapentane 5-butane (M_4_), 1-(1-anthraquinonyloxy) 3, 6 dioxaoctane 9-methane (M_5_) and 1-(1-anthraquinonyloxy) 3 oxapentane 5-methane (M_6_) have been synthesized and characterized by spectral analysis. These ionophores were used in liquid membrane carrier facilitated transport of main group metal cations across supported liquid membrane (SLM). Cellulose nitrate membrane was used as membrane support. Effect of various parameters such as variation in concentration of metal as well as ionophore, effect of chain length and end group of ionophore have been studied. The sequence of metal ions transported by ionophore M_1_ is Na^+^ > Li^+^ > K^+^ > Ca^2+^ > Mg^2+^ and the order of metal ions transported by ionophores (M_2_–M_6_) is Li^+^ > Na^+^ > K^+^ > Ca^2+^ > Mg^2+^. Ionophore M_1_ is selective for Na^+^, Li^+^, and K^+^ and ionophores (M_2_–M_6_) are selective for Li^+^ and Na^+^.

## INTRODUCTION

Carrier-assisted transport through supported liquid membranes is one of the important applications of supramolecular chemistry. The designs of redox-switched crown ethers and lariat ethers have been achieved by researchers [1] in 90s. Crowns are cyclic, introduced by Pedersen [2] in 1967; podands are acyclic, discovered by Vogtle and Angew Chem [3] in 1979; and a new class of crown ethers (combination of cyclic + acyclic) ionophores called lariat crown ethers [4], introduced by Gokel et al. Lariat ethers synthesized for the present study have redox moiety and different chain length of polyethers and have been used as a carrier in facilitated transport of alkali and alkaline-earth metal ions across supported liquid membrane (cellulose nitrate). We have already reported [5] isolation studies of main group (Li^+^, Na^+^, K^+^, Ca^2+^, Mg^2+^) metal ions with redox-switched lariat ethers. The molecular architecture of lariat ether side arm holds the metal ions, and selectivity and carrier ability of redox-switched lariat ethers will be helpful in constructing ion-selective electrodes [6], redox-switchable devices [7], and specific carrier in separation of metal cations. Study of physiological reactions will also be carried out.

## EXPERIMENTAL

### Synthesis of redox-switched lariat ethers

**Scheme 1:**
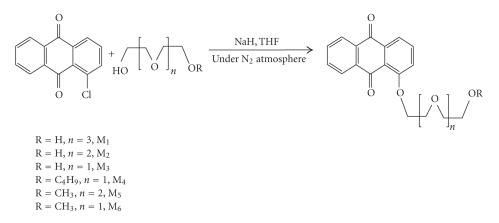


We have synthesized ionophores (M_1_–M_6_) as shown in scheme 1.

#### Preparation of 1-[1-anthraquinonyloxy]-3, 6, 9-trioxaundecane-11-ol (M_1_)-

A solution of tetraethylene glycol (2.89 mL) in THF (10 mL) was added to vigorously stirred suspension of NaH (60% oil dispersion, 0.29 g, and 7.25 mmol) in THF and the mixture was refluxed for 30 minutes. Then a solution of 1-chloroanthraquinone (1.28 g, 5.28 mmol in THF) was added to it and refluxed at 80°C for 10 hours with stirring. This reaction was performed under nitrogen atmosphere. The reaction mixture was concentrated and the residue was mixed with CH_2_Cl_2_ and then washed with water (twice) followed by brine. The organic phase was separated and dried (over MgSO4), filtered, and concentrated. Column chromatography (silica gel, 2% MeOH/CH_2_Cl_2_) followed by recrystallization (CH_2_Cl_2_/hexane then EtOH) gave 2.73 g (80%) of ionophore M_1_ as a yellow solid.

Melting point is 52°C.
IR (KBr) *ν* – 3565(OH), 2940 cm^−1^(CH_2_), 2865 cm^−1^, 1685 cm^−1^(C=O), 1320 cm^−1^, 1270 cm^−1^(ArOCH_2_), 1140 cm^−1^.
^1^H NMR (*δ* in ppm) –3.25 –4.45 (m, 20 H, OCH_2_), 7.20–8.35 (m, 7H, ArH).

Ionophores M_2_, M_3_, M_4_, M_5_, and M_6_ were prepared in the same manner by taking triethylene glycol, diethylene glycol diethylene glycol monobutyl ether, and triethylene glycol monomethyl ether, diethylene glycol monomethyl ether [8], respectively (scheme 1).

### Chemicals

Metal salts as metal picrate (MPic) were prepared as reported earlier [9]. The reagents used in the synthesis of redox-switched ionophores (M_1_–M_6_) were 1-chloroanthraquinone (Lancaster), sodium hydride (Merck Limited, Mumbai, India), and tetraethylene glycol, triethylene glycol, diethylene glycol, diethylene glycol monobutyl ether, and triethylene glycol monomethyl ether, diethylene glycol monomethyl ether (Fluka Chemika-BioChemika, Switzerland). The solvents CHCl_3_, CH_2_Cl_2_, THF (Qualigen, Glaxo India Limited, Mumbai, India) were used as it is.

#### Preparation of membrane

**Figure 1:**
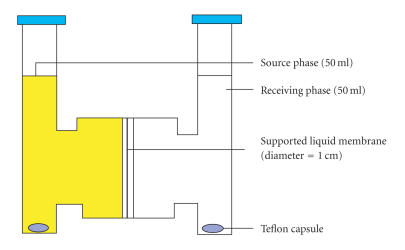
Apparatus for supported liquid membrane transport.

Commercially available synthetic membrane Merck (cellulose nitrate) has been used as a support in SLM studies. The membrane pore size was 0.2 *μ*m. Membranes were impregnated with redox-switched ionophores (M_1_–M_6_), dipped overnight, and used as a membrane support. These impregnated membranes were used for carrier-facilitated transport studies of alkali and alkaline-metal cations. Electron microscope studies are under process (Figure 1).

#### Carrier-mediated transport across supported liquid membrane

Figure 1 shows the apparatus for this study. The supported liquid membrane [10] was positioned between two cylindrical half-cells. One cell compartment (source phase) contained an aqueous solution of the metal salt (50 mL) of 1 × 10^−1^ and the other cell contained the receiving phase (50 mL) double distilled water separated by membrane having an effective diameter of 1 cm. Both phases were stirred with magnetic stirrer at 120 rpm at room temperature, the sample was withdrawn from the receiving phase after 24 hours and analyzed for sample using Systronics flame photometer (Li^+^, Na^+^, K^+^, Ca^2+^) and UV-V is a spectrophotometer for Mg^2+^. Cation flux (*J_M_*) values were calculated by using the relation [11]

(1) $$J_{M}=\frac{C(\text{receiving})V}{\left(At\right)}$$,

where *C* is the concentration of cation in receiving phase in mol/dm^3^, *V* is the volume of receiving phase in dm^3^, *A* is the effective area of membrane in m^2^, and *t* is the time in seconds.

## RESULTS AND DISCUSSION

Transport studies of metal ion across SLM were carried out by ionophores (M_1_–M_6_) using cellulose nitrate membrane as a support. Blank experiments were also carried out for transport studies of metal salts in which membrane was devoid of carrier. No leakage of cation in the membrane was noted. The optimum concentration of metal ion and ionophore was found to be 1× 10^−1^ M and 1×10^−4^ M, respectively (Table 1).

The trend for the transport of cations with ionophore M_1_ is Na^+^ ≫ Li^+^ > K^+^ > Ca^2+^ > Mg^2+^. Ionophore M_1_ having large tetraethylene glycol side chain with anthraquinone moiety shows maximum carrier ability. This is due to their flexible long chain length and additional donor sites for the interaction with all metal (Li^+^, Na^+^, K^+^, Ca^2+^, Mg^2+^) cations. The trend for ionophore M_2_ is Li^+^ > Na^+^ > K^+^. Ionophore M_2_ having triethylene glycol chain shows strong binding affinity with high-charge-density cations, so it forms stable complexes with lithium. Therefore, it shows less transport for K^+^, Na^+^, and no transport for Ca^2+^, Mg^2+^. Ionophore M_3_ having small diethylene chain shows transport for Li^+^ > Na^+^ only because small flexible side arm forms small pseudocyclic cavity by hydrogen bonding [12]. Ionophore M_4_ having butyl group and M_5_, M_6_ having methyl group shows order of transport for metal ion as Li^+^ > Na^+^ > K^+^.

Cation selectivity depends on the particular end group, when the end group is butyl, the ionophore M_4_ binds lithium in comparison to simple –OH group because conformational rigidity of the supporting framework does play a crucial role in binding. The conformation of side chain is such as to enclose the metal cation and there is interaction between coordinating site of the ionophore and the metal ion, as the end methyl group is not too strong. From the results, it is clear that Mg^2+^ cation is not transported in sufficient amount by this ionophore (M_1_–M_6_) due to its highest charge density. Selectivity of ionophore M_4_ is shown at the bottom of Table 1. Ionophore M_6_ having diethylene glycol monomethyl ether shows selectivity for Li^+^ due to cavity fit concept. Ionophore (M_1_–M_6_) shows selectivity towards Li^+^ due to small size and higher charge density of Li^+^ accounts for self-encapsulation [13].

The results inform us that the metal ion transport mainly depends upon the structure of the ionophores like number of donor sites, flexibility of chain length, ionophore concentration, and also on the concentration, charge density, and size of metal cation; and this molecular designing helps in fabrication of redox-switchable devices, molecular wires, as well as chemical sensors.

## Figures and Tables

**Table 1:** Amount of metal cation transported in ppm with redox-switched lariat ethers (M_1_–M_6_) through SLM by using cellulose nitrate membrane as a support. Metal ion concentration −1 × 10^−1^ M, ionophore concentration −1 × 10^−4^ M. Selectivity Li^+^/ Na^+^ 4.8149 (M_4_), % is the percentage of metal ion migration in 24 hours.

Ionophore	Amount of metal ions transported in ppm														

	Li^+^			Na^+^			K^+^			Ca^2+^			Mg^2+^		

	Cation	*J_M_* × 10^−6^	%	Cation	*J_M_* × 10^−6^	%	Cation	*J_M_* × 10^−6^	%	Cation	*J_M_* × 10^−6^	%	Cation	*J_M_* × 10^−6^	%
	transp.	mol/m^2^/s		transp.	mol/m^2^/s		transp.	mol/m^2^/s		transp.	mol/m^2^/s		transp.	mol/m^2^/s	

M_1_	15.00	3.18	37.5	18.91	3.59	47.2	12.32	2.71	49.2	8.63	1.65	21.5	1.13	0.21	2.8
M_2_	8.93	1.92	22.3	3.22	0.81	8.0	3.11	0.78	12.4	—	—	—	—	—	—
M_3_	8.93	1.92	22.3	7.83	1.87	19.5	—	—	—	—	—	—	—	—	—
M_4_	13.53	2.81	33.8	3.12	0.78	7.8	1.13	0.21	4.5	—	—	—	—	—	—
M_5_	6.5	1.66	16.25	5.0	1.23	12.5	3.10	0.61	12.4	—	—	—	—	—	—
M_6_	25	5.21	62.5	13.5	2.61	33.75	2.0	0.41	8.0	—	—	—	—	—	—
